# Anaemia in hospitalised preschool children from a rural area in Mozambique: a case control study in search for aetiological agents

**DOI:** 10.1186/s12887-017-0816-x

**Published:** 2017-02-28

**Authors:** Cinta Moraleda, Ruth Aguilar, Llorenç Quintó, Tacilta Nhampossa, Montserrat Renom, Augusto Nhabomba, Sozinho Acácio, John J. Aponte, Delino Nhalungo, Ariel H. Achtman, Louis Schofield, Helder Martins, Eusebio Macete, Pedro L. Alonso, Clara Menéndez

**Affiliations:** 1 ISGlobal, Barcelona Ctr. Int. Health Res. (CRESIB), Hospital Clínic - Universitat de Barcelona, Barcelona, Spain; 2 Manhiça Health Research Center (CISM), Manhiça, Mozambique; 3 CIBER Epidemiology and Public Health (CIBERESP), Barcelona, Spain; 4 Walter and Eliza Hall Institute for Medical Research, 1G, Royal Parade, Parkville, Victoria, 3052 Australia; 5 Department of Medical Biology, The University of Melbourne, Victoria, Australia; 6 Australian Institute of Tropical Health and Medicine, James Cook University, PO Box 6811, Cairns, QLD 4870 Australia; 7 National Directorate of Health, Ministry of Health, Maputo, Mozambique

**Keywords:** Anaemia, Iron deficiency, Children, Sub-Saharan Africa, Malaria, HIV

## Abstract

**Background:**

Young children bear the world’s highest prevalence of anaemia, the majority of which is of multifactorial aetiology, which in turn hampers its successful prevention. Even moderate degrees of anaemia are associated with increased mortality and morbidity. Despite this evidence, there is a lack of effective preventive programs and absence of consensus in the safety of iron supplementation in malaria areas, which reflects the poor understanding of the contribution of different aetiologies to anaemia. In order to reduce the anaemia burden in the most vulnerable population, a study to determine the aetiology of anaemia among pre-school Mozambican children was performed.

**Methods:**

We undertook a case–control study of 443 preschool hospitalized children with anaemia (haemoglobin concentration <11 g/dl) and 289 community controls without anaemia. Inclusion criteria were: age 1–59 months, no blood transfusion in the previous month, residence in the study area and signed informed consent. Both univariable and multivariable logistic regression analyses were performed to identify factors associated with anaemia and adjusted attributable fractions (AAF) were estimated when appropriate.

**Results:**

Malaria (adjusted odds ratio (AOR) = 8.39, *p* < 0.0001; AAF = 37%), underweight (AOR = 8.10, *p* < 0.0001; AAF = 43%), prealbumin deficiency (AOR = 7.11, *p* < 0.0001; AAF = 77%), albumin deficiency (AOR = 4.29, *p* = 0.0012; AAF = 30%), HIV (AOR = 5.73, *p* = 0.0060; AAF = 18%), and iron deficiency (AOR = 4.05, *p* < 0.0001; AAF = 53%) were associated with anaemia. Vitamin A deficiency and α-thalassaemia were frequent (69% and 64%, respectively in cases) but not independently related to anaemia. Bacteraemia (odds ratio (OR) = 8.49, *p* = 0.004), Parvovirus-B19 (OR = 6.05, *p* = 0.017) and Epstein-Barr virus (OR = 2.10, *p* = 0.0015) infections were related to anaemia only in the unadjusted analysis. Neither vitamin B12 deficiency nor intestinal parasites were associated with anaemia. Folate deficiency was not observed.

**Conclusions:**

Undernutrition, iron deficiency, malaria, and HIV are main factors related to anaemia in hospitalised Mozambican preschool children. Effective programs and strategies for the prevention and management of these conditions need to be reinforced. Specifically, prevention of iron deficiency that accounted in this study for more than half of anaemia cases would have a high impact in reducing the burden of anaemia in children living under similar conditions. However this deficiency, a common preventable and treatable condition, remains neglected by the international public health community.

## Background

Despite significant improvements over the last two decades, global anaemia prevalence in 2010 was 33%, causing 68 million years lived with disability, more than other major causes such as chronic respiratory diseases, with Sub-Saharan Africa being the most affected region [1, 2]. Anaemia increases the risk of child mortality while iron deficiency (ID) has consequences on cognitive and physical development of children [3, 4]. Young children had the highest prevalence in all areas, being this age group the only in which anaemia prevalence increased from 1990 to 2010 [2]. Previous information from Mozambique showed that 74.7% of children between 6 months and 5 years were anemic, having haemoglobin (Hb) ≤5 gr/dl 11.5% of them [5]. Mean Hb concentration showed insignificant differences between regions [5]. In Mozambique anaemia has been reported by the Ministry of Health to be the main nutritional disease since 1996, but no information about the aetiology is available [6, 7].

Despite causing such high levels of disability, the public health community does not pay anaemia the attention it deserves. This lack of prioritisation may be partly due to the habit of treating anaemia as a by-product of other disease processes [3, 8]. Therefore, the World Health Organization (WHO) has emphasised the need of addressing the aetiology of anaemia in different populations [8]. In addition, the WHO guidelines for prevention of ID [3], are rarely implemented due to the commonly held view that iron supplementation increases the susceptibility to some infectious diseases mainly in non-iron deficient children, and the lack of iron status markers in low income settings [9]. With the aim of providing evidence to guide control policies, a study to determine the anaemia risk factors was undertaken in hospitalised pre-school children in rural Mozambique, where the causes of anaemia are poorly understood [6, 7].

## Methods

### Study design

The study was conducted at the *Centro de Investigação em Saúde de Manhiça* (CISM) and the Manhiça District Hospital (MDH), Southern Mozambique. The CISM runs a continuous Demographic Surveillance System (DSS) and round-the-clock morbidity surveillance at the MDH. The area has been described in detail elsewhere [10]. Briefly, malaria was main cause of death in the area (21.8% of cases) followed by pneumonia (9.8%), HIV/AIDS (8.3%) and diarrhoea (8%) [11]. Malaria transmission is perennial with marked seasonality and mainly due to *Plasmodium falciparum* [12]. The HIV prevalence in adults (18–47 years of age) was 37.5% [13] and 1.4% in children ≤11 years old [14]. The HIV-mother-to-child-transmission rates in the first month and at 12 months of age were 9% and 27%, respectively [15]. Almost half (47%) of children visited in the out-patient clinic presented some degree of undernutrition being 6% of them severely malnourished [16].

A case–control study was designed to recruit 450 hospital anaemia cases and 450 community controls. All children with clinical criteria for hospital admission with ages of 1–59 months, no history of blood transfusion in the previous month and residence in the CISM DSS area were offered participation in the study. Hb levels were determined with the Hemocue HB 201^+^ system (Änghelom, Sweden) in children whose guardians signed the informed consent and those with Hb <11 g/dl were recruited as cases. Recruitment was consecutive, from October 2008 to August 2010, running continuously during working hours (8:00–16:00). In order to avoid the confounding effect of malaria seasonality, a maximum of ten cases per week were recruited until reaching 450 cases.

Community controls were randomly selected from the DSS among children between 1 and 59 months of age, and visited at home by project personnel. Hb levels were determined by Hemocue in children with no history of blood transfusion in the previous month whose guardians had signed the informed consent. Those with Hb ≥11 g/dl were invited to a study visit at the MDH. Due to the high prevalence of anaemic children in the community (91%) (unpublished data), only 289 community controls were recruited. Enrolment of cases and controls occurred concurrently.

The recruitment of hospital controls, which would have allowed ruling out possible confounding factors associated with being severely sick, was not possible due to the extremely high prevalence of anaemia among admitted children in this setting. Similarly, due to the high prevalence of anaemia in the community, it was not possible to enrol the planned sample size of controls. The implications of this limitation on the results would depend on the assumptions made as to the proportion of controls exposed to the factor.

The results of the clinical examination and socio-demographic information were entered into standardised questionnaires. Four mL of venous blood was drawn, blood smears and filter paper blood smears were made. A maximum of 1 ml/kg was drawn from children. A stool and a urine sample were collected whenever possible.

### Laboratory methods

#### Haematological studies

Blood counts, different from haemoglobin, were performed using a Sysmex analyser KX21N or XT-2000i (Sysmex Long Grove, IL, USA). Reticulocyte counts were estimated by microscopy on cresil blue-stained blood smears.

#### Biochemical studies

Albumin, prealbumin, and C-reactive protein (CRP) levels were measured with an ADVIA 2400 analyser (Siemens Healthcare, Spain). Folic acid, vitamin B12 and ferritin levels were measured with an ADVIA Centaur (Siemens Healthcare, Spain). sTfR levels were measured in a BN-II nephelometer (Dade-Siemens Healthcare, Spain) and vitamin A levels by reversed phase high-performance liquid chromatography [17]. Erythropoietin quantification was performed using the Quantikine human erythropoietin immunoassay kit (R&D Systems, USA).

#### Bacteriological, virological and parasitological studies

Bacteria were detected in blood cultures in the BACTEC® 9050 (Becton-Dickinson, USA). In children ≤18 months of age HIV infection was defined as a HIV-1 DNA-positive result, detected using the Amplicor HIV-1 DNA-PCR kit (Roche Diagnostics, USA) and in older children it was defined as two positive rapid tests, using the Determine HIV-1/2 Rapid Test (Abbott Laboratories, USA) and confirming by the Uni-Gold Rapid Test (Trinity Biotech Co., Ireland), or as discordant rapid test results resolved by an additional HIV DNA-positive result. Epstein-Barr virus (EBV) and Parvovirus B19 (PV-B19) were identified by real time quantitative PCR (qPCR) using the Artus PCR kits (QIAGEN, Spain). *Plasmodium falciparum (Pf)* parasites were identified by microscopy on Giemsa-stained blood films. *Pf* qPCR was performed for microscopically negative samples. *Schistosoma haematobium* was determined by direct microscopic examination of urine sediment [18]. Intestinal parasites in stool (see Table 3 for details) were detected by microscopic examination using the merthiolate-iodine-formalin concentrations method [19].

#### Genetic studies

Haemoglobinopathies and β-thalassaemia were assessed using the β-thalassaemia Short Program from the Variant Haemoglobin Testing System® (Bio-Rad, Hercules, USA). Detection of α-thalassaemia (3.7 kb deletion) was performed by the GAP-PCR method [20]. Glucose 6-phosphate dehydrogenase (G6PD) deficiency was determined using the Beutler fluorescent spot test [21].

### Definitions

Anaemia was categorised as severe (Hb <5 g/dl) or moderate (Hb ≥5 g/dl to <11 g/dl). Fever was defined as axillary temperature ≥37.5 °C. Bacteraemia was defined as at least one bacterium isolated in blood (excluding common contaminants listed in Table 3). *Pf* infection was defined as the presence of asexual parasites in blood by microscopy or qPCR. Clinical malaria was defined as *Pf* infection plus fever or a history of fever in the preceding 24 h. Detection of parasites by qPCR and a negative blood smear defined submicroscopic *Pf* infections. Hyperparasitaemic *Pf* infection was defined as >100,000 parasites/μl of blood. EBV and PV-B19 infections were defined as detection of the virus in blood by qPCR.

Wasting was defined as a weight-for-height/length Z-score (WHZ) of < −2 standard deviations (SD), stunting as height-for-age Z-score (HAZ) < −2 SD and underweight as weight-for-age Z score (WAZ) < −2 SD [22]. Albumin deficiency was defined as <34 g/l (laboratory reference value), and prealbumin deficiency as <0.142 g/l in children <6 months of age, <0.120 g/l in children 6–12 months of age and <0.108 g/l in children 13–59 months of age [23]. Folate deficiency was defined as <3 ng/ml (laboratory reference value), vitamin A deficiency as <20 μg/dl [24], and vitamin B12 deficiency as <200 pg/ml [25]. ID was defined as the ratio of soluble transferrin receptor to log ferritin (TfR-F index) >1.5 if CRP <1 mg/dl, and >0.8 if CRP ≥1 mg/dl. This biomarker was identified as the best predictor of ID with an accuracy of 71%, using the bone marrow iron content as the gold standard [9]. In order to compare ID prevalence between cases and controls, this ID definition was used instead of bone marrow iron content, as bone marrow examination was undertaken in anaemic children only [9]. Inflammation was defined as a CRP level ≥1 mg/dl. Ineffective erythropoiesis was a Reticulocyte Production Index <2 [26].

### Statistical analysis

Data were double entered using Microsoft Visual FoxPro 5.0 (Microsoft Corp., USA) and analysed using STATA 12 (STATA Corporation, College Station, USA). Crude comparisons were performed by Chi-square or Fisher’s exact tests for proportions, and the Student’s t test or Wilcoxon rank sum test for means or medians, respectively. Multivariable logistic regression analysis was performed by stepwise selection including all variables with *p*-value <0.05 in the crude models, using significance levels of 0.05 and 0.10 for addition to and for removal from the model, respectively. Among the variables with no significant association with anaemia in the univariable model only vitamin B12 and G6PD deficiencies were included in the multivariable model based on biologic plausibility. Intestinal parasitic infections, schistosoma infection, haemoglobinopathy type E, sickle cell trait and β-thalassaemia trait were not included because of their low prevalence. No children in the control group had clinical malaria or hyperparasitemia, so these variables were not included in the regression models. Due to the difficulty in obtaining sufficient volume of blood samples in some children, α-thalassaemia was analysed in only 258 subjects and it was also excluded from the regression analysis. Missing observations were excluded from the multivariate model. Attributable fraction (AF) were estimated using Bruzzi’s approach [27]. The variance of adjusted AF (AAF) was approximated by Taylor series to first derivative terms (delta method) [28].

## Results

### Characteristics of the participants

**Fig. 1:**
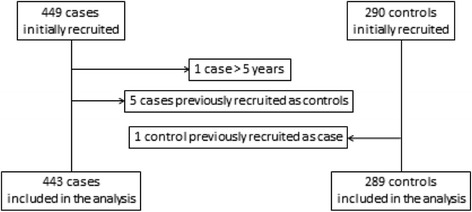
Recruitment flow chart

During the study period 736 children were enrolled. Six children were recruited twice and were included in the first group in which they participated; one case older than 5 years of age was excluded. The analysis was restricted to 443 cases and 289 community controls (Fig. 1). Demographic, clinical and socioeconomic characteristics of participants are shown in Tables 1 and 2.

**Table 1:** Demographic and clinical characteristics of cases and community controls

Characteristic		Study groups		*p*-value
		Cases	Controls	
Gender^1^ (male)		261/443 (59%)	132/289 (46%)	0.0004^2^
Age (months)^3^		19.40 (14.08) [443]	26.61 (18.51) [289]	<0.0001^4^
Jaundice^1^		5/443 (1%)	0/288 (0%)	0.1628^5^
Palpable spleen^1^		96/443 (22%)	1/288 (0%)	<0.0001^2^
Fever^1^		389/443 (88%)	1/289 (0%)	<0.0001^2^
Haemoglobin g/dl^3^		7.88 (1.98) [443]	11.66 (0.75) [289]	<0.0001^4^
Haemoglobin < 5 g/dl^1^		40/443 (9%)	0/289 (0%)	<0.0001^2^
MCV (fl)^3^		71.53 (9.64) [427]	75.63 (7.02) [270]	<0.0001^4^
MCHC (g/dl)^6^		32.60 (4.20) [425]	34.00 (3.30) [270]	<0.0001^7^
RBC distribution width-sd (fl)^6^		45.40 (9.60) [420]	41.75 (6.10) [270]	<0.0001^7^
White Blood Cells (x10^3^/μl)^8^		10.54 (7.54) [426]	8.75 (3.44) [272]	<0.0001^4^
CRP (mg/dl)^6^		5.99 (10.77) [432]	0.05 (0.25) [282]	<0.0001^7^
EPO (U/l)^6^		53.45 (176.10) [434]	10.50 (7.00) [283]	<0.0001^7^
Inflammation^1^		356/432 (82%)	27/282 (10%)	<0.0001^2^
Insufficient erythropoiesis^1^		311/326 (95%)	172/195 (88%)	0.0022^2^
Duration of infant admission (days)^3^		4.93 (4.94) [426]	-	
Outcome of admission^1^	Alive	386/425 (91%)	-	
	Died	13/425 (3%)	-	
	Left	13/425 (3%)	-	
	Transferred	13/425 (3%)	-	

^1^n/N (percentage); ^2^Chi-squared test; ^3^Arithmetic mean (SD) [n]; ^4^t-test; ^5^Fisher’s exact test; ^6^Median (IQR) [n]; ^7^Wilcoxon Rank Sum test; ^8^Geometric mean (SD) [n]

*Abbreviations*: *CRP* C-reactive protein, *EPO* Erythropoietin, *MCHC* Mean cell haemoglobin concentration, *MCV* Mean cell volume, *RBC* Red blood cells

**Table 2:** Socioeconomic characteristics of cases and community controls

Characteristic		Study groups		*p*-value
		Cases	Controls	
Mother’s age (years)^1^		25.59 (6.31) [376]	26.50 (7.03) [281]	0.0826^2^
Number of children^1^		1.73 (1.61) [354]	1.99 (1.85) [270]	0.0549^2^
Marital status^3^	Married	17/432 (4%)	7/279 (3%)	0.4006^4^
	Union	295/432 (68%)	207/279 (74%)	
	Maiden	89/432 (21%)	44/279 (16%)	
	Separate	20/432 (5%)	13/279 (5%)	
	Widow	11/432 (3%)	8/279 (3%)	
Mother was educated^3^		255/440 (58%)	199/287 (69%)	0.0019^4^
Mother works outside home^3^		111/443 (25%)	190/289 (66%)	<0.0001^4^
House of cement^3^		149/443 (34%)	126/289 (44%)	0.0065^4^
Number of rooms^5^		1 (1) [443]	2 (2) [289]	<0.0001^6^

^1^Arithmetic mean (SD) [n]; ^2^t-test; ^3^n/N (percentage); ^4^Chi-squared test; ^5^Median (IQR) [n]; ^6^Wilcoxon Rank Sum test

### Risk factors associated with anaemia

**Fig. 2:**
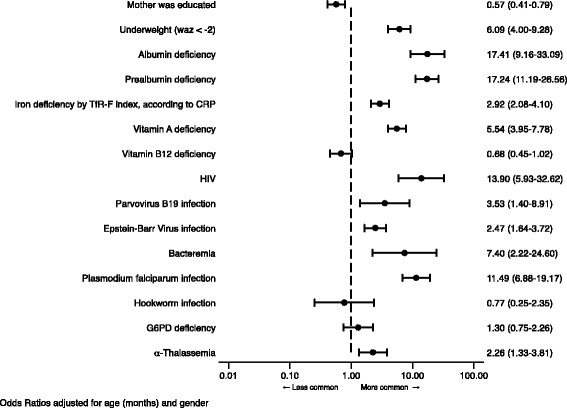
Odds Ratios adjusted for age and gender for factors associated with anaemia, according groups. *Abbreviations*: *CRP* C-reactive protein, *G6PD* Glucose 6 phosphate dehydrogenase, *TfR-F index* ratio of soluble transferrin receptor to log ferritin, *WAZ* Weight for age Z score

The distribution of risk factors associated with anaemia is summarised in Table 3. Adjusted (for age and sex) Odds Ratios (AOR) from the univariable analysis are shown in Fig. 2.

**Table 3:** Distribution of possible aetiological and confounding factors of anaemia among cases and community controls

Variable		Study groups		Total	*p*-value
		Cases	Controls		
Undernutrition					
Underweight (WAZ < −2)^1^		197/443 (44%)	32/284 (11%)	229/727 (31%)	<0.0001^2^
Wasted (WHZ < −2)^1^		134/446 (31%)	18/280 (6%)	152/716 (21%)	<0.0001^2^
Stunted (HAZ < −2)^1^		141/436 (32%)	35/284 (12%)	176/720 (24%)	<0.0001^2^
Albumin deficiency^1^		175/432 (41%)	11/282 (4%)	186/714 (26%)	<0.0001^2^
Prealbumin deficiency^1^		389/430 (90%)	97/280 (35%)	486/710 (68%)	<0.0001^2^
Iron deficiency^1^		263/381 (69%)	111/260 (43%)	374/715 (58%)	<0.0001^2^
Folate deficiency^1^		0/381 (0%)	0/255 (0%)	0/636 (0%)	
Vitamin A deficiency^1^		301/434 (69%)	79/283 (28%)	380/717 (53%)	<0.0001^2^
Vitamin B12 deficiency^1^		68/413 (16%)	53/269 (20%)	121 (18%)	0.2794^2^
Viral infections					
HIV^1^	Negative	297/443 (67%)	266/289 (92%)	563 (77%)	<0.0001^2^
	Positive	93/443 (21%)	6/443 (2%)	99 (14%)	
	Indeterminate	4/443 (1%)	0/443 (0%)	4 (1%)	
	Not done	49/443 (11%)	17/443 (6%)	66 (9%)	
Parvovirus B19 infection^1^		26/436 (6%)	6/287 (2%)	32/730 (4%)	0.0149^2^
EBV infection^1^		117/441 (27%)	39/286 (14%)	156/727 (21%)	<0.0001^2^
Bacteraemia^1^		34/433 (8%)^4^	3/286 (1%)^5^	37/719 (5%)	0.0001^2^
Parasitic infections					
*Plasmodium falciparum* ^1^		179/428 (42%)	21/287 (7%)	200/715 (28%)	<0.0001^2^
Hyperparasitemic *Pf.* ^1^		34/428 (8%)	0/287 (0%)	34/715 (5%)	<0.0001^2^
Submicroscopic *Pf.* ^1^		37/322 (11%)	12/284 (4%)	49/666 (8%)	0.0011^2^
Clinical malaria^1^		172/428 (40%)	0/287 (0%)	172/715 (24%)	<0.0001^2^
Intestinal parasites^1^		6/205 (3%)^6^	10/154 (6%)^7^	16/359 (4%)	0.1051^2^
*Schistosoma haematobium* ^1^		0/172 (0%)	0/175 (0%)	0/347 (0%)	-
Genetic disorders					
G6PD deficiency^1^		44/436 (10%)	22/271 (8%)	66/707 (9%)	0.3805^2^
Haemoglobinopathy^1,8^		4/431 (1%)	2/263 (1%)	6/694 (1%)	1.0000^3^
β-Thalassaemia^1^		1/431 (0%)	4/263 (2%)	5/694 (1%)	0.0711^3^
α-Thalassaemia^1^		77/121 (64%)	59/137 (43%)	136/258 (53%)	0.0010^2^

^1^n/N (column percentage); ^2^Chi-squared test; ^3^Fisher’s exact test; ^4^11 *Streptococcus pneumoniae*, 5 *Staphylococcus aureus,* 5 *Escherichia coli,* 3 *Salmonella typhimurium,* 10 *others.* ^5^1 *E. coli*, 1 *Klebsiella spp* and 1 g negative bacilli, lactose fermenting oxidase negative (these findings were considered contaminations or transient bacteraemia); ^6^the intestinal parasites are 3 *Ascaris lumbricoides*, 2 *Giardia lamblia*, 1 *Strongyloides stercoralis*, ^7^the intestinal parasites are 6 *Ascaris lumbricoides*, 2 *Giardia lamblia*, 1 *Entamoeba hystolitica*, 1 *Strongyloides stercoralis*, ^8^Haemoglobinopathy includes S and E

*Abbreviations*: *EBV* Epstein-Barr virus, *G6PD* Glucose 6 phosphate dehydrogenase, *Pf Plasmodium falciparum*, *WAZ* Weight for age Z score

#### Undernutrition

Cases were more wasted, stunted and underweight than controls (*p* < 0.0001 for all three parameters, Table 3). Deficiencies in albumin and prealbumin were more prevalent in cases than in controls. ID was found in 69% (263/381) of cases and 43% (111/260) of controls (p < 0.0001). ID was more frequent among children with moderate anaemia [70%(241/346)] than those with severe anaemia [63%(22/35)] or in controls [43%(111/260); *p* < 0.0001]. The crude AF of ID was 47%, 95%CI [39, 55] for moderate anaemia and 35%, 95%CI [15, 55] for severe anaemia. Vitamin A deficiency was more frequent in cases, while vitamin B12 deficiency was similar in both groups (*p* = 0.28). Folate deficiency was not found.

#### Bacteraemia

Cases had a higher prevalence of bacteraemia than controls [8%(34/433) vs. 1%(3/286); *p* = 0.0001]. The most commonly isolated bacteria was *Streptococcus pneumoniae* (32%). Among the cases, bacteraemia was similar between children with and without ID [7%(17/258) vs. 8%(9/115); *p* = 0.6649].

#### Viral infections

Cases had a higher prevalence than controls of infections with HIV [21%(93/443) vs. 2%(6/289); *p* < 0.0001], PV-B19 [6%(26/443) vs. 2%(6/287); *p* = 0.0149] and EBV [27%(117/441) vs. 14%(39/286); *p* < 0.0001].

#### Parasitic infections

*Pf* infection was present in 42%(179/428) of cases and 7%(21/287) of controls (*p* < 0.0001). Among the cases, the prevalence of clinical malaria was similar between those with ID [44%(114/258)] and those without it [40%(45/111)] (*p* = 0.5166). Among children with severe anaemia, 71% (25/35) had *Pf* parasitemia compared to 39% (154/393) of those with moderate anaemia (*p* < 0.0001). The prevalence of intestinal parasitic infections was low in both groups [controls 6% (10/154) vs. cases 3%(6/205); *p* = 0.1051)].

#### Erythrocyte genetic disorders

α-thalassaemia was found in 53% of tested children, being more prevalent in cases [64%(77/121) with 16% homozygous (a-/a-) and 48% heterozygous (aa/a-)] than in controls [43%(59/137) with 15% homozygous and 28% heterozygous); *p* = 0.0010]. The frequency of G6PD deficiency was similar in the two groups [cases 10%(44/236) vs. controls 8%(22/271); *p* = 0.3805]. Four children with sickle cell trait but no cases of sickle cell disease were identified. The β-thalassaemia trait was observed in one case and four controls.

#### Multivariable analysis

Age, underweight, deficiencies of prealbumin, albumin and iron, and *Pf* and HIV infections were factors independently associated with anaemia (Table 4). The two main AAF of anaemia were prealbumin deficiency and ID, (77%, 95% CI [69, 86], and 53%, 95% CI, [42, 64], respectively), followed by underweight (43%, 95% CI [39; 48]), and *Pf* infection (37%, 95% CI [33, 41]). The AAF of anaemia associated with HIV infection was 18% (95% CI [13, 22]) (Table 4).

**Table 4:** Adjusted odds ratios from logistic regression model and adjusted attributable fractions for anaemia

Risk factor	Proportion of cases with factor (%)	Adjusted association			AAF		AC
		OR	(95% CI)	*p*-value	Estimate (%)	(95% CI)	
Prealb def.	90.1	7.11	(3.55; 14.24)	<0.0001	77.42	(68.62; 86.23)	235
Underweight	49.2	8.10	(3.82; 17.18)	<0.0001	43.10	(38.54; 47.67)	131
*Pf* infection	42.2	8.39	(3.82; 18.40)	<0.0001	37.21	(33.25; 41.16)	113
Iron def.	70.0	4.05	(2.16; 7.61)	<0.0001	52.71	(41.85; 63.57)	160
Albumin def.	39.6	4.29	(1.78; 10.35)	0.0012	30.37	(22.24; 38.51)	92
HIV	21.5	5.73	(1.65; 19.92)	0.0060	17.71	(13.05; 22.37)	54
*All risk factors in model*					*97.51*	*(96.16; 98.87)*	*295*

*Abbreviations*: *AAF* Adjusted attributable fractions, *AC* Attributable cases, *CI* Confidence interval, def: deficiency, *Pf Plasmodium falciparum*, *Prealb* Prealbumin, *OR* Odds Ratio

The odds ratios presented in this table were adjusted for all the variables included in it. In addition, they were also adjusted for the socio-demographic characteristics that were significant in that multivariable model (age and “mother works outside home”). As these variables were protective, their AAF were not calculated

## Discussion

This paper describes a novel study on the aetiology of anaemia in hospitalised children with varying degrees of anaemia. The findings confirm the multifactorial and overlapping aetiology of anaemia and indicate that undernutrition, ID and malaria and HIV infections are the main factors associated with anaemia. These factors should be prioritised in public health interventions in order to decrease the prevalence of a disease which affects to a 33% of the world population, mainly children in low resource settings [2].

The extremely high prevalence of overall anaemia in children admitted to the MDH (Moraleda et al., unpublished data) highlights the impact of anaemia among children in this area, which prevented the inclusion of a hospitalised control group. The resulting absence of hospital controls and the absence of a case group from the community could result in an overestimation of the effect of diagnoses associated with hospital admission (including HIV, malaria and other infections), which should be taken into account before extrapolate these results to the non-hospitalised population. On the other hand, as it has been reported, hospital controls may provide weaker or null estimates of the associations compared with community controls because of the high frequency of co-morbidities in this group [29]; for example, it is quite likely that hospital controls may not reveal the association of malaria or HIV with anaemia since these are quite frequent conditions among hospitalised children in this setting.

Albumin and prealbumin deficiencies were very prevalent among cases and nearly 50% exhibited underweight. Both proteins are markers of malnutrition, but they are also affected by inflammation [30], explaining why these factors were independently associated with anaemia. In contrast, vitamin A deficiency was not independently related to anaemia. This might be attributable to the association of reduced albumin and prealbumin with vitamin A deficiency [31]. On the other hand, vitamin A deficiency might have been overestimated as vitamin A decreases with inflammation [32]. Alternatively, it may also reflect a poor uptake of vitamin A supplementation programs, suggesting that monitoring of the effectiveness of these programs should be considered a critical addition to public health policies.

While CRP test does serve as a general marker for infection and inflammation, it is not specific enough to diagnose a particular disease. Inflammation, defined by an elevated CRP level, was very common among cases (82%), and thus it was identified as a major risk factor for anaemia. However, this association is difficult to interpret because it actually hides the associations with other underlying factors; for this reason CRP was not included in the multivariable analysis.

In contrast with previous observations in Malawi [33], and similarly to other reports [34], vitamin B12 deficiency was not associated with a risk of anaemia in this study. The general evidence suggests that vitamin B12 deficiency is not a major contributor to anaemia in all settings in Africa.

In this study, folate deficiency was not detected. This may have been underestimated because of difficulties in measuring folate plasma levels [35], or to an increase of folate levels caused by haemolysis during malaria [36]; however, folate levels were also high in children without malaria. Tests with increased sensitivity to measure folate are needed in malaria-exposed populations [35].

The proportion of children with *Pf* parasitemia was increased in those with severe anaemia, suggesting that malaria may contribute more to severe than to moderate anaemia.

Anaemia is one the most frequent signs among HIV-infected children, so the observed association between both conditions was expected [37]. HIV-related anaemia has been mainly associated with uncontrolled HIV-disease, opportunistic infections and nutritional deficiencies, but also to the use of drugs such as zidovudine and cotrimoxazole [38]. At recruitment, the HIV status was known by the relatives in only 44% of the HIV-infected children of the study and from those less than a quarter were on antiretroviral treatment (most of them including zidovudine) and 11% referred to be on cotrimoxazole prophylaxis, both with unknown levels of adherence (data not shown). A direct effect of the HIV as one of the main causes of anaemia was expected, but the lack of the RNA-HIV viral loads results limits this conclusion. Other studied aetiologies, such as undernutrition and bacteriemia, seem to have a role in the anaemia of the HIV-infected children in this study (data not shown).

Prevalences of bacteraemia, PV-B19 and EBV infections were higher in cases than in controls, but no independently related to anaemia. This may be due to the association of these infections with other factors included in the model, such as HIV and malaria [33, 39, 40].

Helminthic infections were rare and not associated with anaemia. These findings, consistent with published data among pre-school children [41, 42] and with studies in this same setting where molecular techniques were used (Mandomando et al. unpublished data), are relevant when considering the target age groups of deworming programs.

In agreement with previous observations in Mozambique, the prevalence of sickle cell trait was very low [43]. On the other hand, more than 50% of those in whom it was tested presented with heterozygous or homozygous α-thalassaemia, indicating that mean cell haemoglobin concentration is not a suitable marker of iron status in populations with a high prevalence of the α-thalassaemia trait [9]. G6PD deficiency was not associated with an increased risk of anaemia despite most cases harbouring infections, the main risk factor to develop anaemia in children with G6PD deficiency [44].

An important fraction of anaemia was due to ID (AAF 53%), indicating that its prevention might have a considerable impact in reducing anaemia prevalence. The association of ID with anaemia was in contrast with data from Malawi where ID was related to a reduced risk of severe anaemia [33]. The authors explain their finding by the observed inverse association between ID and bacteraemia, and the contribution of bacteraemia to anaemia [33]. In the current study, ID was not associated with a reduced prevalence of bacteraemia among cases. The dissimilar observations could be explained by different definitions of study groups, and distinct contributions of ID to anaemia depending on its severity. In the Malawian study, cases were children with severe anaemia and controls were children with Hb ≥5 g/dL, whereas all degrees of anaemia were included in the cases in the study presented here and controls were not anaemic. The protective effect of ID against severe anaemia in the Malawian study was based on a greater prevalence of ID among controls (most of whom had moderate anaemia). This actually concords with the findings in the present study where ID appeared to contribute more to moderate than to severe anaemia, where other factors might play a more important role.

Despite the identification of ID as the 4^th^ greatest risk factor for burden of disease in sub-Saharan Africa [1], its prevention in children exposed to malaria remains unresolved. Discrepancies between the Cochrane review, which concludes that iron supplementation does not adversely affect children living in malaria areas [45], and the WHO guidelines which recommend iron supplementation in these areas only if children have proven to be iron deficient [46], leads to the absence of preventive programs for this significant global health problem.

## Conclusions

In hospitalized Mozambican preschool children, undernutrition, and ID were found significantly associated with anaemia, indicating that their prevention may have a major impact. Malaria and HIV infections were also important contributors to anaemia but the impact of their control would be place specific. These findings should inform the development of future interventional studies (such as those focused on iron supplementation or nutritional improvement), which would then guide the design of effective programs to prevent anaemia, a major killer of young children in developing countries.

## Abbreviations

AAF: Adjusted attributable fractions
AF: Attributable fraction
AOR: Adjusted odds ratio
CISM: Centro de Investigação em Saúde de Manhiça
CRP: C-reactive protein
DSS: Demographic Surveillance System
EBV: Epstein-Barr virus
G6PD: Glucose 6-phosphate dehydrogenase
Hb: Haemoglobin
ID: Iron deficiency
MDH: Manhiça District Hospital
PV-B19: Parvovirus B19
qPCR: real time quantitative PCR
SD: Standard deviations
TfR-F index: Ratio of soluble transferrin receptor to log ferritin
WHO: World Health Organization
